# Positron Emission Tomography in Animal Models of Alzheimer’s Disease Amyloidosis: Translational Implications

**DOI:** 10.3390/ph14111179

**Published:** 2021-11-18

**Authors:** Ruiqing Ni

**Affiliations:** 1 Institute for Biomedical Engineering, ETH & University of Zurich, 8093 Zurich, Switzerland; ni@biomed.ee.ethz.ch; 2 Institute for Regenerative Medicine, University of Zurich, 8952 Zurich, Switzerland

**Keywords:** Alzheimer’s disease, amyloid-beta, animal model, astrocyte, blood–brain barrier, imaging, metabolism, microglia, neuroinflammation, neurotransmitter receptors, positron emission tomography, synaptic density

## Abstract

Animal models of Alzheimer’s disease amyloidosis that recapitulate cerebral amyloid-beta pathology have been widely used in preclinical research and have greatly enabled the mechanistic understanding of Alzheimer’s disease and the development of therapeutics. Comprehensive deep phenotyping of the pathophysiological and biochemical features in these animal models is essential. Recent advances in positron emission tomography have allowed the non-invasive visualization of the alterations in the brain of animal models and in patients with Alzheimer’s disease. These tools have facilitated our understanding of disease mechanisms and provided longitudinal monitoring of treatment effects in animal models of Alzheimer’s disease amyloidosis. In this review, we focus on recent positron emission tomography studies of cerebral amyloid-beta accumulation, hypoglucose metabolism, synaptic and neurotransmitter receptor deficits (cholinergic and glutamatergic system), blood–brain barrier impairment, and neuroinflammation (microgliosis and astrocytosis) in animal models of Alzheimer’s disease amyloidosis. We further propose the emerging targets and tracers for reflecting the pathophysiological changes and discuss outstanding challenges in disease animal models and future outlook in the on-chip characterization of imaging biomarkers towards clinical translation.

## 1. Introduction

Alzheimer’s disease (AD) is the most common cause of dementia, afflicting 50 million people worldwide [1]. AD is pathologically featured by amyloid-beta(Aβ) plaques and neurofibrillary tangles formed by hyperphosphorylated tau, gliosis, neurotransmitter deficits, and neuronal loss leading to cognitive impairment [2]. The abnormal accumulation of Aβ deposits, especially the neurotoxic oligomeric Aβ plays a crucial role in the disease pathogenesis in animal models and in patients with AD [3,4,5,6]. Recent advances in positron emission tomography (PET) using [^18^F]fluorodeoxyglucose (FDG), tracers for Aβ pathology and tauopathy, structural magnetic resonance imaging, and cerebrospinal fluid biomarkers have provided valuable insights into the time course of the pathophysiology of AD continuum, assisted the early and differential diagnosis, and facilitated the development of therapeutics for AD [7,8,9,10,11]. Disease animal models recapitulating AD amyloidosis have been developed including transgenic APP/PS1, APP23, APPswe, J20, PS2APP, arcAβ, 5 × FAD, 3 × Tg mice, TgF344 and McGill-R-Thy1-APP rats [12,13,14,15,16,17,18,19], second-generation App^NL-G-F^, App^hu/hu^ knock-in mice [20,21], third-generation mouse models [22,23], as well as non-human primate model [24]. The animal models accumulate cerebral Aβ pathology, develop gliosis, metabolic and synaptic deficits, and cognitive impairment assessed by behavior tests, and facilitate the understanding of disease mechanisms and the development of treatment strategies. In this review, we focused on the recent development in PET imaging for Aβ, alterations in cerebral glucose metabolism, synaptic neurotransmitter receptors, blood–brain barrier, and neuroinflammation in rodent models of AD amyloidosis.

## 2. Amyloid Imaging 

**Figure 1:**
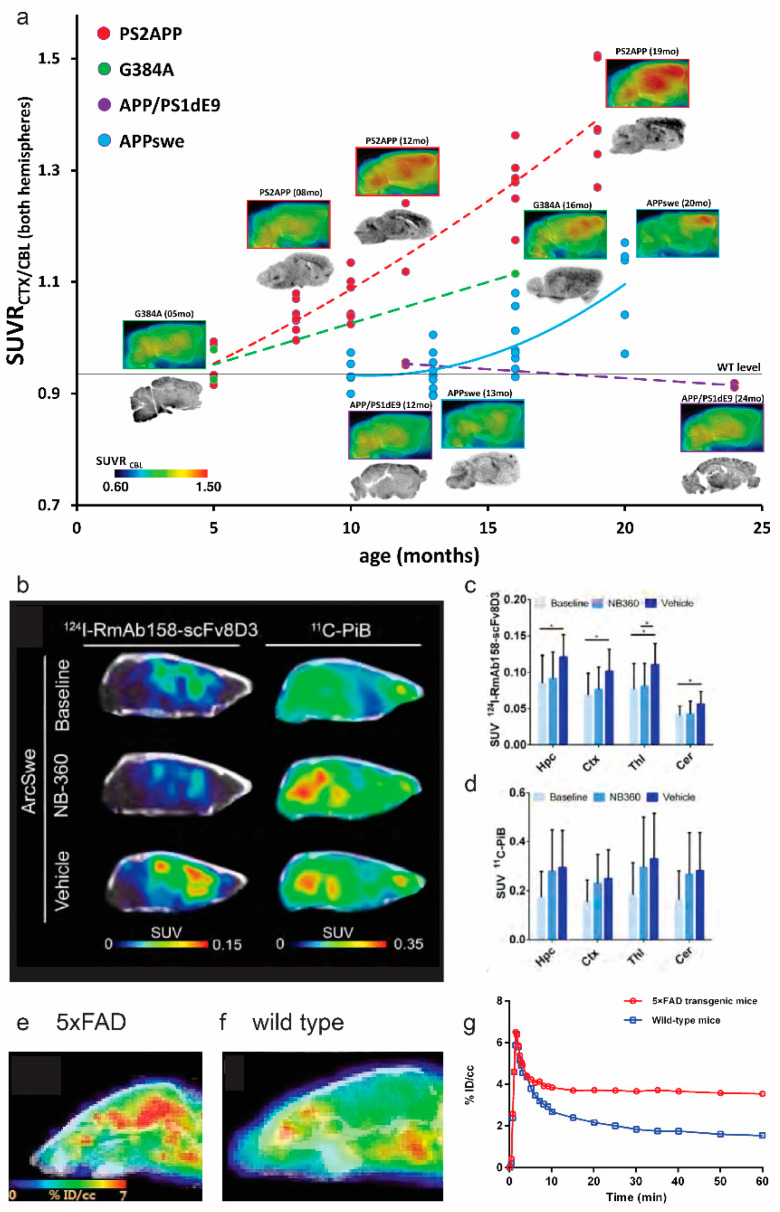
Imaging of amyloid-beta accumulation, and gamma-secretase in amyloidosis animal models of Alzheimer’s disease: (**a**) multi-modal analysis of the four AD mouse strains in cross-sectional [^18^F]florbetaben PET study. Images indicate group averaged sagittal PET slices, normalized to the cerebellum as well as ex vivo autoradiography. Dots indicate PET SUVR cortex/cerebellum in individual mice. Dashed lines express the estimated time-dependent progression in PS2APP, APPswe/PS1G384A, and APP/PS1 mice, fitted with a polynomial function. Reproduced from [49] with permission from PLOS One; (**b**–**d**) PET images and quantification of [^11^C]PiB (40–60 min after injection) and [^124^I]RmAb158-scFv8D3 scans (72 h after injection) expressed as standardized uptake value (SUV): (**b**) comparison of representative [^124^I]RmAb158-scFv8D3 and [^11^C]PiB PET images in ArcSwe animals; (**c**,**d**) quantification of [^124^I]RmAb158-scFv8D3 and [^11^C]PiB in hippocampus (Hpc), cortex (Ctx), thalamus (Thl) and cerebellum (Cer). * *p* < 0.05. Reproduced from [55] with permission from the Society of Nuclear Medicine and Molecular Imaging; (**e**–**g**) PET–CT imaging of γ-secretase in 5 × FAD and wild-type mice; (**e**) PET–CT image of 5 × FAD mice (*n* = 2) and (**f**) wild-type mice (*n* = 2) after i.v. injection of [^11^C]SGSM-15606; (**g**) time activity curve of whole-brain uptake of [^11^C]SGSM-15606 in h and i. Data are expressed as the percentage of injected dose per cubic centimeter (% ID/cc). Reproduced from [59] with permission from Rockefellfigureer University Press.

Ex vivo immunohistochemistry in brain tissues from amyloidosis mouse or rat models has revealed that Aβ pathology initiates first in the cortical region and spreads to the limbic region and finally to the cerebellum [25], in an animal line-dependent manner. A more pronounced load of Aβ deposits was observed in 5 × FAD mice, compared with that in APPswe mice [25,26,27]. In addition to the parenchymal Aβ plaques, cerebral amyloid angiopathy (CAA) is also observed in different amyloidosis animal models, especially in the APPDutch mice, Tg-SwDI, APP/London, APP23, arcAβ, and APPswe mice [28,29,30]. Several Aβ imaging tracers have been developed and applied in animal models of amyloidosis, including benzothiazole derivatives [^11^C]PiB, [^18^F]flutemetamol, [^18^F]florbetaben, [^18^F]FIBT, [^18^F]florbetapir, [^11^C]AZD2184, [^18^F]FC119S and [^18^F]flutafuranol, benzofuran derivatives [^18^F]FACS and [^18^F]FPZBF-2, benzoxazole derivatives [^11^C]BF-227 and [^18^F]MK3328, benzoselenazole derivative [^18^F]fluselenamyl. hydroxyquinoline derivative [^18^F]CABS13, imidazopyridine derivative [^18^F]DRKXH1, as well as [^64^Cu]labelled 8a′–8d and HYR-17 [31,32,33,34,35,36,37,38,39,40,41,42,43,44,45,46,47] (Table 1). Higher cortical amyloid PET tracer uptake was observed in various transgenic or knock-in animal models, compared with wild-type littermates, and validated by the ex vivo immunohistochemical stainings. Longitudinal comparative imaging studies across amyloidosis mouse lines have detected distinct Aβ spreading patterns in vivo. Snellman et al. showed a greater Aβ tracer dynamic range in the brain of the APP23 model, compared with that of APPswe and APP/PS1 models by PET imaging using both [^11^C]PiB and [^18^F]flutemetamol [38,48]. Brendel et al. compared four amyloidosis mouse strains (PS2APP, APPswe/PS1G384A, APP/PS1, APPswe) and found that PS2APP mice demonstrated greater dynamic changes in the longitudinal [^18^F]florbetaben imaging study [49] (Figure 1a). Moreover, comparative studies of amyloid imaging tracers have been performed in a head-to-head manner in animal models, such as comparison among [^11^C]PiB, [^18^F]florbetaben, and [^18^F]FIBT [36], and between [^18^F]florbetaben and [^18^F]flutemetamol [50]; similar patterns of tracer detection of cerebral Aβ distribution in the animal models have been reported in general.

As the commonly used amyloid tracers cannot differentiate parenchymal Aβ plaques and CAA [51], efforts have been made to develop CAA-specific tracers such as resorufin derivatives [52], [^3^H]1, 2 [53]. One of the unsolved questions in Aβ imaging is the detection of small forms of Aβ aggregates. Biechele et al. recently indicated that the non-fibrillar Aβ (positive for 3552 antibodies) significantly impacted the [^18^F]florbetaben PET signal, in addition to the Thiazine red-stained fibrillar Aβ, in App^NL-G-F^ and APP/PS1 mice from 3–12 months of age [54]. In addition to the small chemical dyes, PET using Aβ antibodies conjugated to a transferrin receptor antibody such as [^124^I]RmAb158-scFv8D3 and [^124^I]8D3-F(ab’)2-h158 have been developed to detect cerebral accumulation of small forms of Aβ. These tracers harbor an improved blood–brain barrier permeability and have been demonstrated in several transgenic mouse models of amyloidosis. Meier et al. demonstrated that the uptake of [^124^I]RmAb158-scFv8D3 and [^124^I]8D3-F(ab’)2-h158 was significantly higher in the cortical regions of transgenic ArcSwe mice, compared with non-transgenic littermates. In addition, the distribution pattern of PET using [^124^I]8D3-F(ab’)2-h158 differs from that by PET using [^11^C]PiB in the brain of tg-ArcSwe mice, indicating a preference to different types of Aβ by these two tracers (Figure 1b–d) [55]. Given the quantitativeness of in vivo microPET, non-invasive imaging using [^18^F]florbetaben and [^18^F]florbetapir for Aβ load have been applied for longitudinal monitoring of the treatment effect in animal models, such as using γ-secretase modulator and β-secretase 1 inhibitor [56,57,58]. Xu et al. recently demonstrated using [^11^C]SGSM-1560 for in vivo detection of an increased level of γ-secretase in 5 × FAD, compared with wild-type mice [59] (Figure 1e–g).

**Table 1:** Amyloid-beta PET imaging in animal models of Alzheimer’s disease amyloidosis.

Tracer	Animal Model	References
[^11^C]PiB	APPswe mice	[37,48,60]
	5 × FAD mice	[61]
	APP/PS1 mice	[36,48,62,63,64,65,66]
	3 × Tg mice	[67]
	APP23 mice	[33,48,68]
	Aged non-human primates	[69,70]
[^18^F]florbetapir, AV-45	5 × FAD mice	[61,71]
	TASTPM mice	[72]
	APP/PS1 mice	[58,73]
[^18^F]florbetaben, AV-1	PS2APP mice	[49,74]
	APPswe mice	[49,75]
	App^NL-G-F^ mice	[54,74,76,77,78]
	APPswe/PS1G384A mice	[49]
	APP-SL70 mice	[74,79]
	TgF334 rats	[80]
	APP/PS1 mice	[49,54,66,81]
[^11^C]AZD2184	APPswe mice	[82]
	APP/PS1 mice	[83]
[^18^F]flutafuranol AZD4694, NAV4694	McGill-R-Thy1-APP rats	[43]
	APPswe mice	[42]
[^18^F]flutemetamol	APP23, APPswe, APP/PS1 mice	[37,38]
[^18^F]FIBT	APP/PS1 mice	[36]
[^18^F]FC119S	5 × FAD, APP/PS1 mice	[34,35]
[^18^F]FACT, [^11^C]BF-227	APP/PS1 mice	[84,85]
[^18^F]fluselenamyl	APP/PS1 mice	[86]
[^124^I]RmAb158-scFv8D3	Tg-ArcSwe, App^NL-G-F^ mice	[55]
[^124^I]8D3-F(ab’)2-h158	Tg-ArcSwe, APPswe mice	[87]
[^18^F]CDA-3	5 × FAD mice	[88]
[^64^Cu]HYR-17	5 × FAD mice	[39]
[^64^Cu]8a’–8d	5 × FAD mice	[44]
[^18^F]DRKXH1	APP/PS1 mice	[40]
[^18^F]CABS13	APP/PS1 mice	[41]

[^11^C]AZD2184, 2-(6-[^11^C]methylaminopyridin-3-yl)-1,3-benzothiazol-6-ol; [^11^C]BF-227, [^11^C]2-(2-[2-Dimethylaminothiazol-5-yl]ethenyl)-6-(2-[fluoro]ethoxy)benzoxazole; [^18^F]CABS13, 2-[^18^F]fluoroquinolin-8-ol; [^18^F]CDA-3, [^18^F]croconium dye for amyloid; [^18^F]DRKXH1, 5-(4-(6-(2-[^18^F]fluoroethoxy)ethoxy)imidazo[1,2-alpha]pyridin-2-yl)phenyl; Fab, antigen-binding fragment; [^18^F]FACT, 2-[(2-{(E)-2-[2-(dimethylamino)-1,3-thiazol-5-yl]vinyl}-1,3-benzoxazol-6-yl)oxy]-3-[^18^F]fluoropropan-1-ol; [^18^F]FIBT, 2-(p-methylaminophenyl)-7-(2-[^18^F]fluoroethoxy)imidazo-[2,1-b]benzothiazole; [^18^F]FC119S, 2-[2-(N-monomethyl)aminopyridine-6-yl]-6-[(S)-3-[^18^F]fluoro-2-hydroxypropoxy]benzothiazole; [^18^F]florbetaben, 4-[(E)-2-[4-[2-[2-(2-[^18^F]fluoranylethoxy)ethoxy]ethoxy]phenyl]ethenyl]-N-methylaniline; [^18^F]florbetapir, 4-[(E)-2-[6-[2-[2-(2-[^18^F]fluoranylethoxy)ethoxy]ethoxy]pyridin-3-yl]ethenyl]-Nmethylaniline; [^18^F]fluselenamyl, (Z)-5-(2-(5-(2-[^18^F]fluoroethoxy)benzo[d][1,3]selenazol-2-yl)vinyl)-N,N-dimethylpyrimidin-2-amine; [^18^F]flutafuranol, 2-[2-[^18^F]fluoro-6-(methylamino)-3-pyridinyl]-1-benzofuran-5-ol; [^18^F]flutemetamol, 2-[3-[^18^F]fluoro-4-(methylamino)phenyl]-1,3-benzothiazol-6-ol; [^11^C]PiB, Pittsburgh compound B, 2-[4-([^11^C]methylamino)phenyl]-1,3-benzothiazol-6-ol; scFv, single chain fragment variable.

## 3. Cerebral Glucose Metabolism Imaging

**Figure 2:**
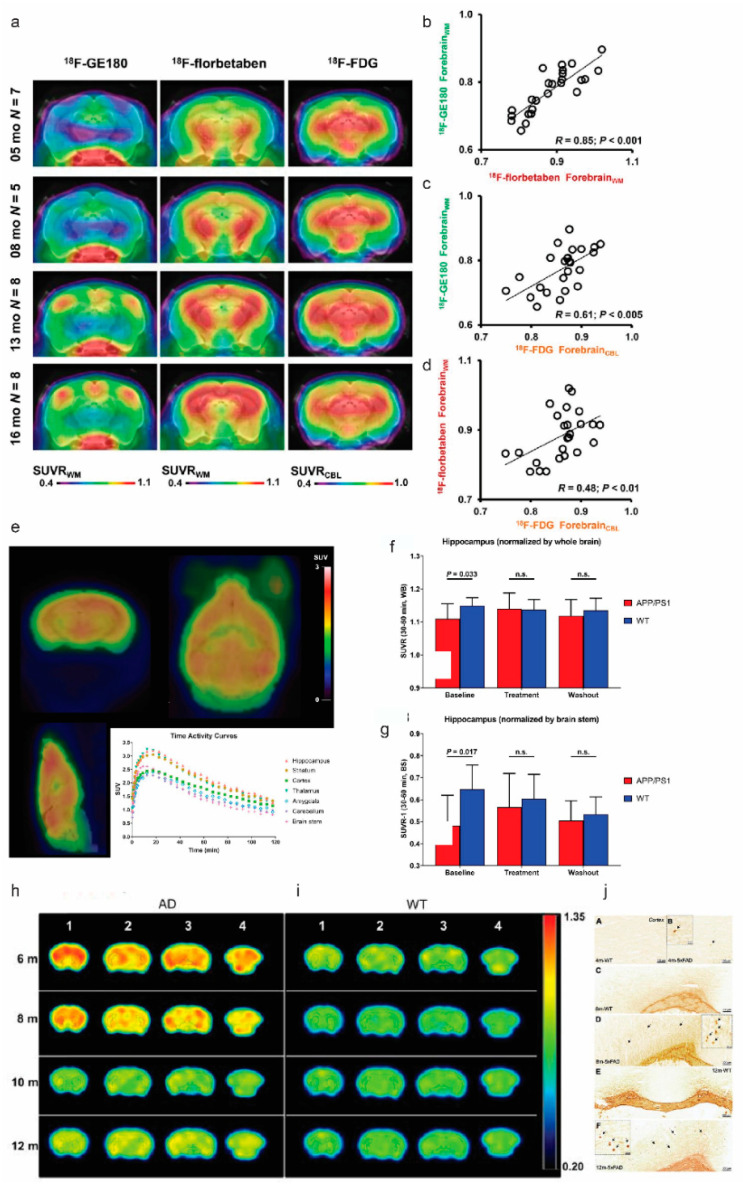
In vivo imaging of translocator protein, cerebral glucose metabolism, synaptic density, and butyrylcholinesterase, in amyloidosis animal models of Alzheimer’s disease: (**a**–**d**) [^18^F]GE-180, [^18^F]florbetaben, and [^18^F]FDG PET imaging at different ages of PS2APP animals; (**a**) coronal planes of mean SUVR maps projected on an MRI mouse atlas (grayscale); (**b**–**d**) correlations between the different forebrain radiotracer SUVR for all PS2APP mice. Reproduced with permission [134] with permission from the Society of Nuclear Medicine and Molecular Imaging; (**e**–**g**) representative [^11^C]UCB-J PET image and time-activity curve in APP/PS1 mice; (**a**) static SUV image (30–60 min after injection) overlaid on atlas brain MR image; (**b**,**c**) hippocampal SUVRs in wild-type and APP/PS1 mice during baseline, treatment, and washout phases: whole-brain SUVR (**b**) and brain stem SUVR (**c**). Reproduced from [113] with permission Society of Nuclear Medicine; (**h**–**j**) PET images for BChE imaging in 5 × FAD mice; (**c**,**d**) axial view of PET images in 5 × FAD and wild-type mice after i.v. administration of [^11^C]4 at different ages; (**e**) Staining for BChE enzymatic activity in 4-, 8-, and 12-month-old brains of wild-type (**A**,**C**,**E**) and 5 × FAD mice (**B**,**D**,**F**) using the Karnovsky–Roots method. BChE staining showed an increase in enzyme activity in the cerebral cortex of 5 × FAD at different ages in comparison with wild-type (**A** to **F**) mice. Magnified images show the co-occurrence of plaques with BChE enzyme activity in different regions of the cerebral cortex (**B**,**D**,**F**) Reproduced from [121] with permission from Ivyspring International Publisher.

Brain glucose dysregulation plays an important role in AD [89]. Post-mortem studies reported higher levels of brain tissue glucose concentration, lower levels of glucose transporter 3, and glycolytic flux in the brain from patients with AD, compared with controls, associating with the severity of AD pathology [89]. [^18^F]FDG PETs have been routinely used for detecting the reduced cerebral glucose metabolism (CMRglc) in disease-specific brain regions in patients with AD, Frontotemporal dementia, and Parkinson’s disease to improve the diagnostic accuracy [9,90]. In lab settings, [^18^F]FDG PET have been assessed along with Aβ imaging in various amyloidosis rodent models such as APPswe mice, 5 × FAD, APP/PS1, 3 × Tg, Tg4-42, TASTPM mice, and McGill-R-Thy1-APP rats [43,66,71,91,92,93,94,95] (Table 2) (Figure 2a)**.** However, [^18^F]FDG uptake is known to be highly sensitive to experimental conditions such as anesthesia and handling, as well as genotype, age, and gender of the animal models [96]. Most of the studies in rodent amyloidosis models reported a global reduction in CMRglc, although few exceptions of increased CMRglc (associating with gliosis) were also reported [61]. A recent study by Xiang et al. further showed that microglial activation states drive glucose uptake and [^18^F]FDG-PET alterations [97].

**Table 2:** PET imaging in of neurotransmitter receptors, blood–brain barriers, enzymes, metabolism, and synaptic density in animal models of Alzheimer’s disease amyloidosis.

Target	Tracer	Animal Model	References
CMRglc	[^18^F]FDG	3 × Tg mice	[94,98,99,100,101,102],
		APPswe mice	[92]
		APP/PS1 mice	[58,66,72,103,104,105,106]
		Tg4-42 mice	[91,107]
		5 × FAD mice	[61,71,81,108,109]
		3 × Tg rats	[110]
		APP23 mice	[111]
		McGill-R-Thy1-APP rats	[43]
		TASTPM mice	[72,112]
		Aged monkey	[70]
SV2A	[^11^C]UCB-J	APP/PS1 mice	[113]
		ArcSwe, Tg-L61 mice	[114]
	[^18^F]SynVesT-1	APP/PS1 mice	[115]
mGluR5	[^18^F]FPEB	5 × FAD mice	[116,117]
		APP/PS1 mice	[118]
	[^11^C]ABP688	Tg-ArcSwe mice	[119]
α7nAChR	[^11^C]MeQAA	Aged monkey	[69]
	[^18^F]ASEM	TgF334 rats	[80]
AChE	[^11^C]MP4A	APP23 mice	[120]
BChE	[^11^C]4	5 × FAD mice	[121]
GABAR	[^11^C]flumazenil	APP23 mice	[120]
GSM	[^11^C]SGSM-1560	5 × FAD mice	[59]
IIa HDAC	[^18^F]TFAHA	3 × Tg mice	[122]
GLP-1R	[^18^F]FBEM-Cys^39^-exendin-4	3 × Tg mice	[102]
D_2_R	[^18^F]fallypride	3 × Tg, 5 × FAD mice	[102,117]
MC1	[^18^F]BCPP-EF	Aged monkey, SAMP10 mice	[69,70,123,124]
Copper	[^64^Cu]GTSM	TASTPM mice	[125]
MT	[^11^C]MPC-6827	J20 mice	[126]
GSK3β	[^11^C]OCM-44, [^3^H]PF-367	APPswe mice	[127]
	[^11^C]2	3 × Tg mice	[128]
RAGE	[^11^C]FPS-ZM1	APPswe mice	[129]
ABCC1	[^11^C]BMP	APP/PS1 mice	[130]
ABCG2	[^11^C]erlotinib	APP/PS1 mice	[131]
P-GP ABCB1	[^11^C]tariquidar	APP/PS1 mice	[131]
	[^11^C]metoclopramide	APP/PS1 mice	[132]
	(R)-[^11^C]verapamil	APP/PS1 mice	[133]

ABC: ATP-binding cassette transporter; α7 nAChR: α7 icotinic acetylcholine receptor; AChE, acetylcholine esterase; BChE: butyrylcholinesterase; CMRglc: cerebral metabolic rate of glucose; D2: dopamine receptor D2; [^18^F]FDG: [^18^F]fluorodeoxyglucose; GABAR: gamma-aminobutyric acid receptor; GLP-1R: glucagon-like peptide-1 receptor; GSK3β: glycogen synthase kinase-3b; GSM: γ-secretase modulator; IIa HDAC: class IIa histone deacetylases; LPS: lipopolysaccharide; MC1: mitochondrial complex 1; mGluR5: metabotropic glutamate receptor type 5; MT: microtubule; NP: nanoparticle; P-GP: P-glycoprotein; SV2A: synaptic vesicle glycoprotein 2A.

## 4. Synaptic and Neurotransmitter Receptor Deficits

### 4.1. Synaptic Vesicle Glycoprotein 2A

Synapse loss is reported in the post-mortem frontal cortex of patients with AD, correlating with cognitive severity [135]. Synaptic vesicle glycoprotein 2A (SV2A) is located at the synapses across the entire brain and is the binding site for the antiepileptic drug levetiracetam [136]. SV2A involves in vesicle trafficking exocytosis and is crucial for neurotransmission and postnatal brain development [137]. Mendoza-Torreblanca et al. suggested that SV2A either regulates the presynaptic Ca^2+^ levels during repetitive activity or is a target for residual Ca^2+^. Higher loads of cerebral Aβ deposits have been reported in the brain of SV2A knock-out mice, compared with control littermates [138]. A 40% reduction in SV2A signal by PET using [^11^C]UCB-J was observed in the hippocampus in patients with AD, compared with cognitively normal control cases [139,140]. Kong et al. showed that SV2A over-expression was associated with the downregulation of β-site APP-cleaving enzyme 1 and apolipoprotein E genes, indicating that SV2A impacts Aβ production. However, Nowack et al. showed that overexpression of SV2A increased synaptic levels of the calcium-sensor protein synaptotagmin, resulting in a neurotransmission deficit [141]. Thus, modulation of SV2A as a potential treatment requires careful dosing and close monitoring of the SV2A levels. Several SV2A PET imaging tracers have been developed including [^11^C]UCB-J, [^18^F]UCB-H [142], [^18^F]SynVesT-1 [143], [^18^F]SDM-8 [144], and [^18^F]MNI-1126 [145] (Table 2). PET measures of Aβ deposition were found associated with regional synaptic density measured by [^11^C]UCB-J in patients with early AD [139,146]. Few studies have reported on SV2A imaging in AD animal models. Bertoglio et al. demonstrated that [^11^C]UCB-J is bound specifically to SV2A in mouse brain and that the radioligand binding can be quantified by kinetic modeling using an image-derived input function [147]. Toyonaga et al. showed that in vivo [^11^C]UCB-J detected reduced levels of SV2A in APP/PS1 mice and the treatment effects of tyrosine kinase Fyn inhibitor Saracatinib in mitigating the [^11^C]UCB-J reduction [113] (Figure 2e–g). Xiong et al. recently compared the [^11^C]UCB-J binding in tg-ArcSwe and wild-type mice [114] and did not observe a clear difference between the two groups. [^18^F]SynVesT-1, [^18^F]analog of [^11^C]UCB-J, has demonstrated favorable in vivo brain uptake in non-human primate [148]. Sadasivam et al. showed a lower [^18^F]SynVesT-1 standard uptake value (SUV) across the whole brain of APP/PS1 mice, compared with non-transgenic mice [115]. The results from a static (30–60 min post-injection) [^18^F]SynVesT-1 PET scan were found comparable to kinetic modeling results [115].

### 4.2. Glutamate Receptors

The glutamate receptors are classified into the N-methyl-D-aspartate receptor (NMDAR), α-amino-3-hydroxy-5-methyl-4-isoxazolepropionate (AMPA)-kainate receptor, and metabotropic glutamate receptors (mGluRs). The glutamate receptors mediate excitatory neurotransmission, involve in multiple second messenger systems, and are essential in learning and memory [149,150]. Glutamate excitotoxicity and disruption of the glutamate receptor-mediated normal signaling are implicated in AD [151,152]. Aβ reduces glutamatergic transmission and inhibits synaptic plasticity [153,154]. Direct interaction between Aβ oligomers and glutamate receptors including NMDAR [155], mGluR subunit mGluR5 [156], AMPA receptor subunit GluA3 [157], and GluA1 [158] have been demonstrated, leading to impaired synaptic plasticity in the animal models [159]. Chronic pharmacological inhibition of mGluR5 has been shown to prevent cognitive impairment and reduce pathological development in APP/PS1 mice [160]. Thus, glutamate receptors have been important targets for AD therapeutics. Several imaging tracers for glutamate receptors have been developed, including [^11^C]K-2 [161] and [^11^C]HMS011 [162] for AMPA receptor, [^18^F]GE-179 [163] and [^18^F]PK-209 for NMDAR [164], [^11^C]Me-NB1 [165] for NMDAR GluN1/GluN2B subunits [166], as well as [^18^F]FPEB, [^11^C]ABP688, and [^18^F]PSS232 for mGluR5 [167,168,169]. In patients with AD, PET using [^18^F]FPEB [170] and [^11^C]ABP688 [171] revealed consistent reductions in regional mGluR5 binding in the hippocampus and amygdala, compared with non-demented controls. Sofar only mGluR5 imaging has been reported in amyloidosis animal models and showed conflicting results probably due to different animal models utilized (Table 2). Lee et al. demonstrated an age-dependent 35% decrease in the level of [^18^F]FPEB measures of mGluR5 in the cortical and subcortical brain areas in 5 × FAD mice at 9 months of age, compared with 3 months of age, validated by ex vivo assessment of mGluR5 protein expression levels [116]. However, Varlow et al. showed that [^18^F]FPEB uptake increased in the brain of 10-month-old APP/PS1 mice, compared with controls [118]. Fang et al. reported similar levels of [^18^F]FPEB uptake in the brain of Tg-ArcSwe mice, compared with control mice at different ages [119]. However, immunoblotting results indicated that the level of mGluR5 in Tg-ArcSwe mouse brain lysate was higher, compared with control mice, at 12 months of age, not at 8 and 16 months of age [119]. Further studies are needed to elucidate the dynamic alteration in glutamate receptors in AD animal models.

### 4.3. Cholinergic System

The cholinergic system is essential for learning, memory formation, attention, and regulating inflammation [172]. The cholinergic system includes nicotinic acetylcholine receptors (nAChR), muscarinic acetylcholine receptors (mAChR), acetylcholinesterase (AChE), and butyrylcholinesterase (BChE). α7 nAChR and α4β2 nAChR are the most abundant nAChR subtypes in the brain. The cholinergic system is impaired early in AD associated with the cognitive, behavioral, and global functioning decline [172,173,174]. Reduced basal forebrain cholinergic neurons, increased levels of α7 nAChR [175,176], and reduced levels of M1 mAChR [177] were reported in the cortical regions of post-mortem brain from AD patients, compared with control. Interaction between α7 and α4β2 nAChR and different forms of Aβ aggregates have also been reported [178,179,180,181]. Several recent PET tracers, including [^11^C]NS14492 [182], [^11^C](R)MeQAA [69], and [^18^F]ASEM for α7 nAChR [183], [^11^C](+)3-MPB [184] and [^18^F]fluorobenzyl-dexetimide [185] for mAChR, [^11^C]LSN3172176 [186] for M1 mAChR, and [^11^C]MK-6884 for M4 mAChR [187] have been developed (Table 2). PET using [^11^C]nicotine imaging showed that the cortical nAChR binding correlated with the cognitive function of attention in patients with mild AD [188]. Few in vivo PET studies for the cholinergic system have been performed in AD models. Nishiyama et al. demonstrated higher [^11^C](R)-MeQAA brain uptake in the thalamus, hippocampus, striatum, and cortical regions, along with increased [^11^C]PiB detection of Aβ load and impaired [^18^F]BCPP-EF binding to mitochondrial complex 1 in the brain of aged monkey [69]. Chaney et al. demonstrated lower levels of [^18^F]ASEM in TgF334 rats, compared with wild-type at 18 months of age [80]. Rejc et al. recently reported increased levels of BChE along with Aβ accumulation using [^11^C]4 and [^18^F]florbetaben, respectively, in brain of 5 × FAD mice at 4–12 months of age, compared with wild-type mice [121] (Figure 2h–j). In comparison, comparable levels of AChE were observed in APP23, compared with wild-type mice at 10–13 months of age, assessed by PET using [^11^C]MP4A [120].

## 5. Blood–Brain Barrier

**Figure 3:**
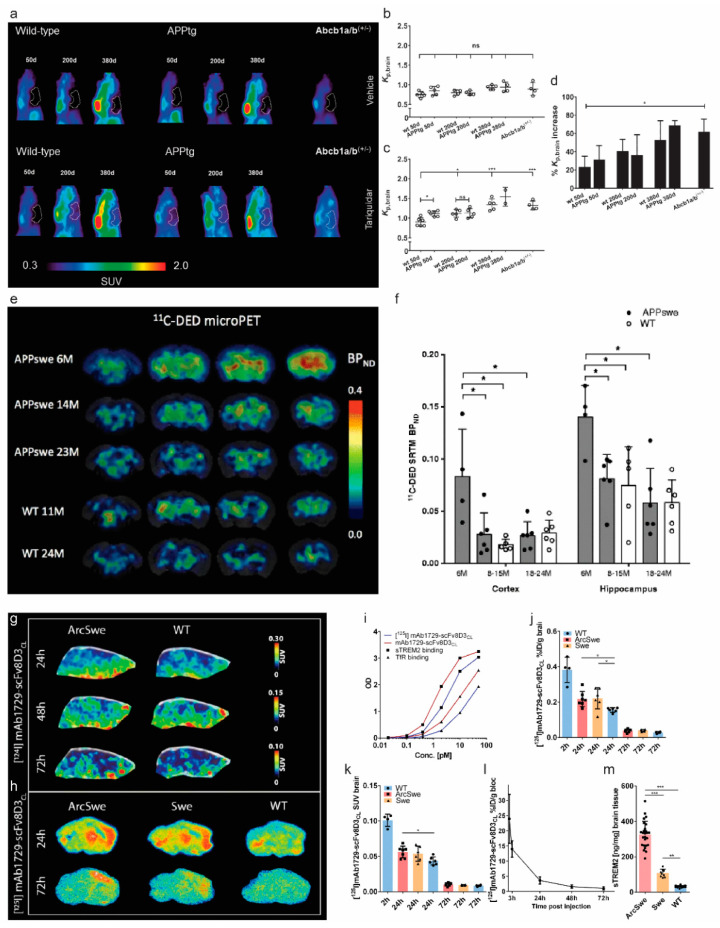
In vivo imaging of blood–brain barrier, astrocytosis, and triggering receptors expressed on myeloid cells (TREM) 2 in amyloidosis animal models of Alzheimer’s disease: (**a**–**d**) imaging of P-glycoprotein (P-gp, ABCB1) using (*R*)-[^11^C]verapamil; (**a**) sagittal PET summation images (0–60 min) of wild-type and APP/PS1 mice aged 50, 200 and 380 days and Abcb1a/b^(+/−)^ mice pre-treated i.v. with vehicle (**b**) or tariquidar (4 mg/kg) (**c**) at 2 h before start of the PET scan. The whole-brain region is highlighted as a white line. In (**d**), the mean percentage increase in Kp, brain of individual tariquidar-treated animals relative to mean Kp, brain value of vehicle group is shown. Ns: not significant, * *p*  <  0.05, *** *p*  <  0.001. Reproduced from [133] from Sage Publication; (**e**,**f**) [^11^C]deuterium-l-deprenyl ([^11^C]DED) microPET imaging in APPswe and wild-type (WT) mice: (**e**) [^11^C]DED microPET coronal parametric BP_ND_ maps images; (f) [^11^C]DED binding in the cortex and hippocampus, expressed as BP_ND_, obtained from simplified reference tissue model of [^11^C]DED using the cerebellum as a reference region, in three groups of APPswe mice aged 6-, 8–15, and 18–24 months and two groups of wild-type mice aged 8–15 and 18–24 months. Significant differences between groups are indicated by * *p*  <  0.05. Reproduced from [82] with permission from Springer Nature; (**g**–**m**) PET imaging of triggering receptor expressed on myeloid cells 2 (TREM2) level in ArcSwe, Swe, and wild-type mice; (**g**) representative SUV scaled sagittal PET images with [^124^I]mAb1729-scFv8D3CL at 24 h, 48 h, and 72 h after injection; (**h**) radioligand distribution in brain tissue displayed in sagittal ex vivo autoradiography images in ArcSwe, Swe, and wild-type animals at 24 h and 72 h after injection (**h**); (**i**) binding comparison of [^125^I]mAb1729-scFv8D3CL and unlabelled mAb1729-scFv8D3CL by using ELISA. Percent of injected dose (**j**) and SUV (**k**) of [^125^I]mAb1729-scFv8D3CL in brain 2 h, 24 h, and 72 h after injection; (**l**) level of [^124^I]mAb1729-scFv8D3CL in blood, which was sampled 1 h, 3 h, 24 h, 48 h, and 72 h after injection; (**m**) TREM2 levels in TBS extracted brains of ArcSwe, APPSwe, and wild-type mice at the age of 18 months. Reproduced from [205] with permission from Springer Nature.

Blood–brain barrier (BBB) is impaired at an early disease stage in AD [189,190]. Whether the BBB dysfunction is secondary to Aβ pathology or a causal factor has not been fully elucidated. In amyloidosis animal models of AD, BBB disruption is observed in mouse models such as arcAβ and APP/PS1 but not prevalent in certain mouse lines such as the PS2APP line [191,192]. Several receptors presented in the BBB have been explored as PET imaging targets, such as adenosine triphosphate-binding cassette (ABC) transporter ABCC1, ABCG2, ABCB1 (P-glycoprotein, P-gp), and receptor for advanced glycation endproducts (RAGE). P-gp plays an important role in the clearance and efflux of Aβ from the brain into the blood across the brain endothelial luminal membrane [193]. The levels of P-gp expression and activity were found to be decreased in the brains of AD patients, compared with that in control cases, as well as in the APP mouse model, compared with wild-type mice [194]. Several P-gp tracers such as (R)-O-[^18^F]fluoroethylnorverapamil, (R)-N-[^18^F]fluoroethylverapamil, (R)-[^11^C]verapamil, [^11^C]tariquidar, [^11^C]metoclopramide, and [^18^F]MC225 have been developed [130,131,132,133,195,196,197,198] (Table 2). Zoufal et al. demonstrated an age-dependent reduction in the cerebral P-gp function in APP/PS1 mice, compared with wild-type mice assessed by PET using (*R*)-[^11^C]verapamil [133] (Figure 3a–d) and by using [^11^C]metoclopramide [132].

However, (*R*)-[^11^C]verapamil showed suboptimal brain uptake, and further improvement and evaluation of P-gp function using novel tracers with improved properties are needed. In addition, PET using 6-bromo-7-[^11^C]methylpurine ([^11^C]BMP) showed an increased level of ABCC1 along with [^11^C]PiB detection of an increased level of Aβ pathology in the brain of APP/PS1 mice, compared with wild-type mice [130]. The increase in the ABCC1 level has been assumed to be related to the upregulation of its expression in astrocytes as a protective mechanism. Imaging of ABCG2 by PET using [^11^C]erlotinib has been reported in APP/PS1 mice: no alteration in the level of ABCG2, compared with wild-type mice, was observed [131].

Receptor for advanced glycation end products (RAGE) is a BBB transporter and a binding site for advanced glycation end products and mediates Aβ transportation across the BBB into the brain [199,200]. The expression level of RAGE was found increased in post-mortem AD brains, compared with that in control cases [199]. RAGE tracers such as [^11^C]FPS-ZM1 [201], [^18^F]RAGER [202], [^18^F]InRAGER [203], and [^64^Cu]Rho-G4-CML nanoparticle (multimodal) have been developed [204]. The only imaging study conducted in the AD animal model by Luzi et al. showed that [^11^C]FPS-ZM1 uptake in the brain of APPswe was similar, compared with that of wild-type mice [129]. Further development and studies are needed to evaluate RAGE imaging tracers in AD animal models and in patients with AD.

## 6. Neuroinflammation Imaging

Several recent articles have provided thorough reviews on neuroinflammation PET imaging in AD patients and AD animal models [206,207,208,209,210,211]. Thus, here, we discuss briefly the recent development in neuroinflammation imaging in AD amyloidosis animal models. Neuroinflammation plays an important role in the pathogenesis of AD and appears early in the development of the disease [212,213,214]. Microglia are the resident macrophages in the central nervous system, engulf Aβ plaques, and are important for maintaining brain homeostasis [214,215]. Recent single-cell sequencing and transcriptomics have demonstrated a transcriptionally distinct and neurodegeneration-specific profile of microglia termed disease-associated microglia (DAM) [216,217,218]. The 18 kDa translocator protein (TSPO) located on the outer mitochondria membrane of microglia has been the most investigated target for microgliosis PET imaging. Three generations of TSPO tracers have been developed with improved properties: the first-generation (R)-[^11^C]PK11195 [219]; the second-generation [^11^C]PBR28 [220], [^18^F]FEDAA1106 [68], [^18^F]DPA-714 [105]; the third-generation [^18^F]GE-180 [134] (Figure 2a) and [^11^C]ER176 [221]. PET using various 18 kDa translocator protein (TSPO) tracers have demonstrated an early microgliosis preceding the Aβ deposition in several animal models of amyloidosis including APP23, hAPP-J20, APPSL70, App^NL-G-F,^ and PS2APP mice [76,77,215,222,223,224,225]. Sacher et al. showed an asymmetry and hemispheric predominance of Aβ accumulation detected by using [^18^F]florbetaben accompanied by microglial activation assessed by using [^18^F]GE-180 in five mouse lines, including APP/PS1, PS2APP, APP-SL70, APPswe transgenic mice, and App^NL-G-F^ knock-in mice [74,226]. Due to the diverse cellular location of TSPO expression on astrocytes and endothelial cells, in addition to that on microglia, tracers specific for microglial expression and of the disease-associated profile are of high interest [227,228,229]. Emerging targets and tracers include [^11^C]SW125M139 for purinergic P2X7 receptor [230,231], [^124^I] mAb1729-scFv8D3CL for triggering receptors expressed on myeloid cells (TREM) 2, [^11^C]AZD1283 for purinergic P2Y12 receptor [232], [^11^C]CPPC [233] and [^11^C]GW2580 [234] for colony-stimulating factor 1 receptor, [^11^C]KTP-Me for cyclooxygenase 1 [235] have been reported in AD animal models. Meier et al. showed a higher expression level of triggering receptor expressed on myeloid cells 2 (TREM2) in the brain from ArcSwe mice, compared with wild-type mice at 24 h, 48 h, and 72 h after injection by autoradiography using [^124^I] mAb1729-scFv8D3CL [205] (Figure 3g–m). The tracers for purinergic P2Y12 receptor [232] show a more specific microglial localization and thus are of high potential.

## 7. Discussion

In vivo longitudinal imaging in animal models of AD amyloidosis has provided valuable insights on the spatiotemporal links between different pathophysiology. A range of molecular imaging tracers for neuroinflammation, synaptic density, and neurotransmitter receptor deficits have been developed and provided a comprehensive picture of AD [11,210,236,237]. In addition to the aforementioned targets, many emerging targets show potential as indicators for pathological alterations in AD and are yet to be further investigated in amyloidosis animal models. These include (1) microgliosis; (2) astrocytosis; (3) metal dysregulation and copper trafficking, e.g., using [^64^Cu]GTSM [125]; (4) reactive oxygen species [238] and pH alterations [239]; (5) microtubule using [^11^C]MPC-6827, [^11^C]HD-800, [^11^C]WX-132-18B [126,240,241]; (6) sigma 1 receptor using [^11^C]HCC0929, [^18^F]FTC-146, [^18^F]IAM6067 and [^11^C]SA4503 [242,243,244]; (7) mitochondria imaging using [^18^F]BCPP-EF [123]; 8) glycogen synthase kinase-3 imaging using [^11^C]2, [^11^C]OCM-44, [^3^H]PF-367 [128,245].

Among the aforementioned emerging microgliosis tracers, the tracers for purinergic P2X7 receptor [230,231], P2Y12 receptor [232] are of high interest due to their specific cellular location on microglia. In addition, astrocytes are essential for maintaining the homeostasis, synaptic plasticity, and inflammatory response in the central nervous system [246] and play key roles in the onset and progression of AD. Reactive astrocytes show disease-associated profiles and exert dynamic functions (neuroprotection and neurotoxicity) in AD [247,248,249,250,251]. Few studies have been reported on PET imaging of astrocytosis in AD animal models. PET using irreversible monoamine oxidase B (MAO-B) inhibitors [^11^C]deuterium-L-deprenyl (DED) showed an early astrocytosis preceding the Aβ accumulation assessed by using [^11^C]AZD2184 in the brain of APPswe at 6 months of age, compared with wild-type mice (Figure 3e,f). A similar finding of an early increase in [^11^C]DED binding was reported in Tg-ArcSwe mice, compared with wild-type littermates [252]. Several novel MAO-B tracers have been developed including [^11^C]SMBT-1 [253] based on *(S*)-[^18^F]THK5117 structure [254] and [^18^F]6 [255]. In addition, a novel astrocytic tracer [^11^C]BU99008, which targets imidazoline-2 binding sites (I2BS), has shown specific and high-affinity binding properties in post-mortem characterization [256] and demonstrated promising results in the recent in vivo PET studies in patients with AD [257,258].

Several earlier studies have reported the complicated temporal and spatial association between [^18^F]FDG, TSPO, and amyloid accumulation: reduced [^18^F]FDG uptake, increased Aβ deposition using [^11^C]PiB or [^18^F]florbetaben [64,134], and increased microglial activation using [^18^F]GE-180 [134] (Figure 2a–d), and [^18^F]DPA-714 has been reported in animal models [105]. Tsukada et al. reported reduced [^18^F]FDG uptake, increased [^11^C]PiB measures of Aβ deposition, increased [^11^C]DPA-713 for microglia activation, and reduced [^18^F]BCPP-EF for mitochondrial complex 1 in the brain of aged monkeys [70]. Given the recent finding of microglial [^18^F]FDG-PET uptake [97], further studies may potentially use [^18^F]FDG-PET for monitoring the microglial status in treatment targeting at microglia. In addition, markers that can specifically reflect synaptic and neuronal function are needed. Amyloidosis animal models show cortical, hippocampal atrophy, and enlargement of ventricle assessed by using structural magnetic resonance imaging, although to a less extent, compared with that in tauopathy animal models [259,260]. Multi-modal imaging [261] or multi-tracer imaging studies combining microgliosis, [^18^F]FDG, and SV2A imaging to provide more comprehensive functional and molecular readouts are thus highly desired [262].

The challenges in bridging the translational gaps of PET imaging in rodent models and in patients with AD may include (1) different rodent models of AD demonstrated divergent time courses and patterns of pathophysiological development. Thus, rational selection of optimal animal models and age for investigation is thus critical in PET imaging studies in tracer evaluation [263]; (2) in addition, species difference in cell types, protein expression level, available binding sites, and post-translational modification of the target added to the complexity [264]. For example, the Aβ deposits formed in the APP mouse models and in aged primates are structurally different from that in the brain from patients with AD [265]. Thus, models that better recapitulate the human AD pathology will greatly boost the AD research, such as the Aβ-KI mouse modeling late-onset AD [23] and the third-generation mouse model [22]; databases of comprehensive deep phenotyping in disease animal models such as “MODEL-AD” by the Alzheimer Consortium Think Tank [266,267] (www.model-ad.org/, accessed on 15 October 2021) are instrumental in facilitating the translational research. Systems biology approaches, including single-cell sequencing, transcriptomics, biochemical characterization, and behavioral assessments, along with in vivo imaging data, will provide accurate interpretation of the readouts [268].

## 8. Conclusions

We provided an overview of PET imaging in animal models of AD amyloidosis, highlighting recent development in visualizing Aβ, cerebral glucose metabolism, synaptic and neurotransmitter receptor deficits, BBB impairment, and neuroinflammation, and proposed outstanding challenges for future development to increase the translational power of preclinical PET in AD.

## Data Availability

All data are contained within the article.
